# Needs analysis for an undergraduate dental curriculum in KPK, Pakistan: Gap identification and general needs assessment

**DOI:** 10.12669/pjms.40.5.8364

**Published:** 2024

**Authors:** Bushra Mehboob, Usman Mahboob, Brekhna Jamil, Neelofar Shaheen

**Affiliations:** 1 Dr. Bushra Mehboob. BDS, FCPS. Assistant Professor, Department of Oral and Maxillofacial Surgery, Peshawar Dental College, Peshawar, Pakistan. Riphah International University, Islamabad, Pakistan; 2 Dr. Usman Mahboob. MBBS, MPH, FHEA, DHPE. Associate Professor, Institute of Health Professions Education & Research, Khyber Medical University, Peshawar, Pakistan; 3 Dr. Brekhna Jamil. BDS, MPH, MHPE. Associate Professor, Institute of Health Professions Education & Research, Khyber Medical University, Peshawar, Pakistan; 4 Dr. Neelofar Shaheen MBBS, MHPE. Assistant Professor, Department of Health Professions Education, Peshawar Medical College, Peshawar, Pakistan. Riphah International University, Islamabad, Pakistan

**Keywords:** Curriculum, Students Dental, Need Analysis, Pakistan, Undergraduate

## Abstract

**Objective:**

Dental institutes continue to face challenges in making the transition from a discipline-based to an integrated curriculum. The need analysis is often the first step in the development and implementation of any curriculum. This study intends to carry out a needs analysis for a contemporary dental curriculum in private and public sector dental colleges of Peshawar, KPK, Pakistan.

**Methods:**

The mixed method study was carried out at public and private dental institutes in Peshawar from April to July 2022. To guarantee triangulation, data were gathered from three sources. The first source was an analysis of the Pakistan Medical and Dental Council’s dental curriculum accreditation standards. The second source was gathering the experts’ perspectives, and the final source was a systematic literature search to explore the necessity for an integrated undergraduate dental curriculum from the experiences and expertise of contemporary curricula.

**Results:**

Thematic analysis identified the need for the dental curriculum to be a five-year BDS program, involvement of students, and inclusion of digital dentistry and environmental sustainability in the dental curriculum. PMDC accreditation standards focus on alignment in mission, vision, curricular outcomes, an integrated curriculum, and a quality assurance system for assessment. Experts identified the need for a patient-centered curriculum focusing on integrated patient care. They also identified that the current educational environment should be improved to sustain a contemporary dental curriculum in Pakistan. For the literature review, nine articles were included in the final review

**Conclusion:**

The current dental curriculum is not accommodating to the needs of the students in Peshawar. The current dental education environment lacks the infrastructure, logistics, and teacher training to sustain the standards set by PMDC.

## INTRODUCTION

Undergraduate dental education is facing multiple challenges today. There are many new ideas, advancements, and trends in medical education that have been approved all over the world.^1^ Medical colleges in Khyber Pakhtunkhwa, Pakistan are in a transition to shift to an integrated medical curriculum whereas according to the Pakistan Medical Commission survey, 66% of dental colleges and universities are still following traditional curriculum.^2^ In this traditional model, basic dental sciences are taught in the first two years of the dental curriculum with little or no clinical integration, while clinical subjects are taught, and clinical training is done in the next two years. This discipline-wise model with compartmentalization, results in unnecessary replication and is confusing for the students.^3^,^4^

Dental education is an expanding field that is constantly changing and redesigning. Every day we are seeing newly emerging patterns of diseases and newer methods to better solve oral problems. In this era of a rapid paradigm shift, the dental curriculum should also reflect flexibility to better cater to the dental needs of people.^5^

Keeping in view the rapidly changing and evolving disease patterns, it is necessary to train students in an integrated manner where cognitive-affective dissonance is reduced to a minimum. Internationally, more dental institutes are shifting towards an integrated approach to help produce graduates who are prepared to provide patient-centred care in a timely and efficient manner.^6^

There are limitations to the discipline-wise model of dental curriculum from the students’ perspective. Early years tend to focus on memorization, with fewer directives to develop psychomotor skills. Theoretical knowledge can also lead to cognitive overload.^7^

Moving from a discipline-based model to an integrated curriculum model is faced with distinct administrative, and logistic challenges and it requires careful assessment, planning, and analysis as a first step.^8^ This study intends to carry out a needs analysis of a dental undergraduate curriculum that can be implemented in private and public sector dental colleges of Peshawar, KPK, Pakistan.

## METHODS

The needs analysis was done in the public and private sectors of Peshawar by following the first two steps of Kern’s six-step approach to Curriculum design.^9^

### Ethical Approval

It was obtained from Khyber Medical University reference no: 1-11/IHPER/MHPE/KMU/22-25 obtained on 20^th^ March 2022.

A mixed method study design was used. The following sources were considered for needs analysis.

- Literature Search
- Published report on dental curriculum by Pakistan Medical and Dental Council
- Opinion of Experts.

### 1. Literature Search

**Fig.1:**
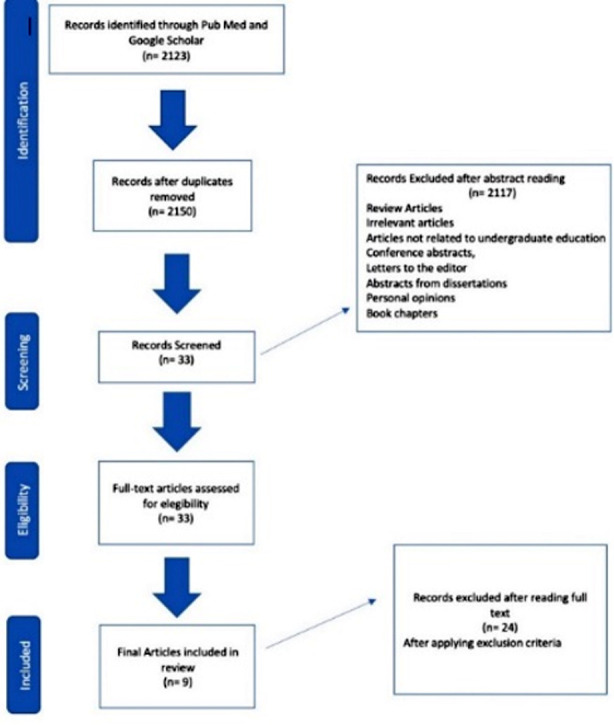
Preferred Reporting Items for Systematic Reviews and Meta-Analyses extension for Scoping Reviews flow chart.

The literature search was carried out on “the needs of dental graduates in the 21st century” from 15-20th May 2023. Sources used were PubMed and Google Scholar by using the following search terms: “contemporary”, “dental education”, “21st century”, “dental graduates”, and “needs”. We limited our search to articles published in English between 2019 and 2023. Nine articles were selected in the final review (Fig.1). The detail of the articles is given in Table-I.

**Table-I:** Critical appraisal of selected articles

S. No	Author, Date	Aim	Study Design	Location	Participants	Major findings
1.	Sheikh GM 10 2022	To explore the perceptions of the dental faculty regarding the changes required with regard to subjects, the teaching methodology, assessment, and innovative recommendations in Pakistan.	Qualitative Research	Pakistan	13 dental faculty members Semi-structured one-to-one interview	Globalization of curriculum and innovative teaching strategies are required. The curriculum should be community oriented. Inclusion of forensic odontology and mentorship. The program duration should be 5 years
2.	Kornegay EC 11 2021	To describe faculty perspectives on the curriculum needs and vision of future graduates	Qualitative Research	USA	80 faculty members Focused group Discussion	A collaborative patient care, integrated curriculum, and student involvement are essential. The 2040 graduate should be a leader and exhibit resilience in response to changing environment.
3.	Leon et al.^12^ 2018	To re-inspect geriatric dentistry education and recognize its curricular content	Quantitative	USA	56 dental schools	51.7% of schools had some form of compulsory undergraduate clinical education in geriatric dentistry.
4.	Lone et.al,^13^ 2017	To develop and assess a novel cranial nerve animation as a supplemental learning aid for dental students.	Quantitative	Ireland	Development of animation and use on first-year dental students, graduate entry dental students (year 1) and dental hygiene students (year 1)	The use of the animation can act as a supplemental tool to improve student knowledge of the cranial nerves
5.	Ali K^14^ 2022	To explore experiences and perceptions of students and staff regarding decolonization of the curriculum in a dental undergraduate programme.	Descriptive Cross sectional	England	34 staff members and 120 students	Minority Ethnic students were, when compared with white counterparts, less likely to report that their programme included opportunities for group discussions about privilege and need better representation.
6.	Kelly N^15^ 2023	To introduce a local framework for the identification of patients at higher risk of suicide and the management of suicidal disclosures during dental outpatient appointments	Descriptive Cross sectional	England	30 students surveyed	Awareness and training related to mental and suicide health must be introduced to undergraduate curricula.
7.	Seminario AL^16^ 2020	To pilot the effectiveness and acceptance of a new global oral health curriculum	Interventional Study	USA	80 students	An integration of the Global Health project in dental curriculum has a positive impact on graduates.
8.	Poblete P^17^ 2020	To identify topics (knowledge and skills) from the dental curricula that would benefit from having a 3D learning resource	Mixed Method	UK	Seven focus groups and three Interviews after which Survey constructed and sent to final year dental students, newly dental graduates and academics from three Scottish universities	97 topics identified for 3D technology development. In survey, detailed anatomy of the temporomandibular joint, dental anesthesia, dental clinical skills techniques, dental occlusion and mandibular functioning were top priorities.
9.	Duane B^18^ 2021	To: 1.Inform educators about the main principles of sustainable clinical practice 2.Report initial discussion on the importance of embedding sustainability within the domains and major competences of the Graduating European Dentist curriculum	Descriptive Cross sectional	Berlin	Questionnaire circulated to potential participants at ADEE sustainability workshop	The declining state of planetary health is impacting population health significantly. Environmental sustainability is affected by dental treatments and should be included in existing curriculum of dental education.

### 2. Published report on dental standards guidelines by Pakistan Medical & Dental Council

Pakistan Medical and Dental Council is the accreditation/regulatory body in Pakistan. It published guidelines on standards for undergraduate dental curriculum.^19^ Thematic analysis of the document was done to understand the standards required for medical and dental colleges for accreditation. Only the standards related to students and curriculum were analysed for this study.

### 3. Opinion of experts

A total of 34 faculty members involved in teaching dentistry to undergraduate dental students for more than three years were recruited via a purposeful sampling technique to take their opinions on the academic needs of dental graduates. The researcher’s judgemental bias was addressed by developing consensus by the team and strictly adhering to agreed-upon eligibility criteria. Faculty members, who did not have an additional certificate or master’s degree in health professions education were excluded from the study. For this purpose, a ten-item questionnaire was designed. We used a two-step validation procedure to make sure our research instrument was valid. Three professionals in the field of medical education first reviewed the questionnaire, offering insightful comments and recommendations for improvement. Later on, a pilot study with ten dental education experts who were the faculty of basic or clinical dentistry was carried out.

For the pilot survey, the questionnaire was sent to 10 faculty members. After the initial pilot phase and final approval by the experts, a formal survey was carried out through Google Forms. Links were sent to the participants along with the informed consent form through emails and WhatsApp. Data were collected from April to July 2022. The data was entered and analyzed by SPSS version 26 and presented as frequencies and percentages.

## RESULTS

Out of a total of 34 participants, 55.9% (n=19) were male while 44.1% (n=15) were female, 50% (n=17) had more than 10 years of, and 26.5% (n=9) had five to ten years of teaching experience, while 23.5% (n=8) had less than five years of teaching experience. Moreover, 24 teachers (70.6%) belonged to clinical specialties and 10 (29.4%) were from basic dental science subjects.

The survey questions along with faculty responses are given in Table-II, while the comparison of results from the literature search and analysis of PMDC standards for accreditation is given in Table-III.

**Table-II:** Survey questions along with faculty responses.

Question	Response ; n=34	
In your opinion, are the dental graduates/house officers prepared to handle patients independently?	Yes (91.2%)	
	No (8.8%)	
If not, where do you see the deficiency?	Knowledge (0)	
	Skill (20.7%)	
	Attitude (2.9%)	
	All of these (67.6%)	
	Not applicable (8.8%)	
What in your opinion is/are the reason for the deficiency in students handling patients independently?	Non-Structured Curriculum	Yes: 23.5% No: 76.5%
	Ineffective teaching strategies	Yes: 23.5% No: 76.5%
	Less emphasis on clinically relevant topics	Yes:23.5% No:76.5%
	Lack of clinical application of the knowledge	Yes: 61.8 % No: 38.2 %
	Lack of students interest	Yes: 20.6% No: 79.4%
	Wrong assessment strategies	Yes: 17.6% No: 82.4%
What in your opinion should be the goals of a dental graduate?	To be knowledgeable and skilful: 29.4%	
	Prepared to handle patients: 29.4%	
	Life-long learners: 8.9%	
	Professional Competence with Ethical values: 14.7%	
	Efficient: 2.9%	
	Community service: 2.9%	
	Clinical application of knowledge: 2.9%	
	No Response: 8.9%	
During your last year of teaching, mention the areas/topics of your specialty that should have been included in more detail?	None: 47.1%	
	Diagnosis, Treatment planning and clinical application: 11.8%	
	Advanced dental and prosthetic procedures: 5.9%	
	Majority topics left in modular system: 5.9%	
	Maxillofacial Oncology: 5.9%	
	Pain and periodontal surgeries: 5.9%	
	Restorative materials and preventive orthodontics: 5.9%	
	Slide preparation: 2.9%	
	Behavioral and social sciences: 2.9%	
	Complete Denture: 2.9%	
	Exodontia: 2.9%	
What difficulties did you face during teaching?	Lack of student interest, attention and understanding: 47.1%	
	No difficulty: 29.4%	
	Class duration and timings: 11.8%	
	Lack of clinical application: 2.9%	
	Lack of facilities: 8.8%	
Which of these teaching strategies would like to include in your teaching?	Didactic Lectures	Yes: 5.9% No: 94.1%
	SGD	Yes: 17.6% No: 82.4%
	PBL	Yes: 58.8% No: 41.2%
	CBL	Yes: 50% No: 50%
	TBL	Yes: 44.1% No: 55.9%
What technologies would you like to incorporate into your teaching?	None: 47.1%	
	Technology for preclinical skill development: 8.8%	
	3-D technology: 20.6%	
	Gamification: 8.8%	
	Innovative teaching strategies: 8.8%	
	Audio Visual Aids: 5.9%	
What in your opinion are the barriers to effective teaching and learning in the current learning environment?	Lack of financial incentives for teachers	Yes: 52.9% No: 47.1%
	Lack of infrastructure and logistics	Yes: 61.8% No: 38.2%
	Lack of training for teachers	Yes: 47.1% No: 52.9%
	Lack of curricular planning	Yes: 41.2% No: 58.8%
Kindly give your suggestions to improve the learning experience of the students	None: 20.7%	
	Community Service: 5.9%	
	Better assessment strategies: 2.9%	
	Clinical Practice: 11.8%	
	Co-curricular activities: 2.9%	
	Student’s encouragement, interaction and feedback: 11.8%	
	Faculty Development: 14.7%	
	Improved logistics: 5.9%	
	Innovative teaching strategies and use of technology: 8.8%	
	Mental health prioritization: 2.9%	
	Integrated curriculum: 5.9%	
	Improved admission criteria for students: 2.9%	
	Increase teaching hours: 2.9%	

**Table-III:** Comparison of literature review and PMDC guidelines.

Area	Source 1 literature review	Source 2 published guidelines by accrediting body PMDC^19^
Curricular organization	Involvement of students and faculty in curricular reforms	Institutional Autonomy, but compliance with PMDC standards
		Alignment with vision, mission, and curricular outcomes
	Community-oriented curriculum	Patient-centred and outcome-based
		Integrated curriculum
Program Duration	Five Years	Four Years
Curricular Content	Should Include Technological advancements, special care, forensic odontology, Geriatric Dentistry and Environmental sustainability	Decided in consensus with subject experts and medical educationist. Inclusion of social sciences, study skills, and leadership.
Curricular Outcomes	Graduates should be able to apply evidence in local context and understand global health challenges.	Outcomes should be relevant, clearly communicated, and continuously reviewed.
Achieving Dental graduates’ preparedness to handle patients independently	Patient centred collaborative care.	An integrated form of curriculum with clearly defined Learning outcomes
	Globalization of Dental curricula	Humanities and elective rotations
	Decolonization	Student centred instructional tools
		Early clinical exposure and Community orientation
Instructional Strategies	3-D Learning Resources in Dentistry	Active learning strategies
		Early clinical exposure with structured and supervised learning in clinical environment
		Innovative educational strategies quality standard
Assessment	Not found	Balance of formative and summative assessment
		Quality assurance system in place
		Standard setting and integrated assessment methods
Required improvements in Educational Environment	Mentorship Program	Student selected components
	Mental health and suicide awareness programs	Student financial support program, counselling service and Student’s feedback

## DISCUSSION

This paper uses an innovative three-step methodology to identify the needs required to transform the current dental education system. In this study, 91.2% of experts thought dental graduates were unprepared to handle patients independently. Most experts (61.8%) stated the reason was a lack of clinical application of knowledge followed by less emphasis on clinically relevant topics, and a non-structured curriculum. The literature and survey suggest a structured curriculum with the involvement of students in curricular reforms.^10^ However, the current situation in Pakistan suggests that most dental colleges and universities in Pakistan still follow traditional or subject-based curricula (66%).^2^

The program duration in the dental curriculum was identified to be five years. The duration of the dental course is an important consideration as it affects the amount of time and resources required to train dental professionals. The duration varies from four to five years in different countries. A shorter duration may result in limited exposure to clinical training. Conversely, a longer duration may increase students’ financial burden and delay their entry into the workforce.^20^

The curricular content of an undergraduate dental course should be relevant and practical. An integrated curriculum will strive to organize similar subjects together such as oral biology integrated with anatomy and physiology. Worldwide the subject of dentistry is advancing. Thus, the content should cover various areas, including technological advancements^17^, special care and geriatric dentistry^21^, clinical communication skills,^22^ and environmental sustainability.^18^

The literature suggests using 3-dimensional learning resources in dentistry, student-centered strategies, and technologies in TLM. The experts in this study identified a lack of interest by students as the major obstacle in teaching. This means that the dental curriculum is not catering to the needs of the students of the 21^st^ century. The curriculum should include joint sessions of basic and clinical subjects, early clinical exposure, and pre-clinical skills lab rotation so the students can learn basic skills in a safe environment.^23^

Guidelines by PMDC have different basic and quality standards for the undergraduate dental curriculum. However, the local needs of students demand better logistics and teacher training to implement the standards. The majority of the experts (68.8%) suggested a lack of logistics and infrastructure as a deficiency in the current learning environment. Similar results were found by Sethi A. on the learning environment in dental colleges of KPK, where faculty members reported a lack of energy and enthusiasm in the academic atmosphere.^24^ In contrast to this, the literature suggests mental health, well-being, and wellness be a priority in the dental curriculum. Ethnic minority students should be given representation.^14^ Moreover awareness about mental health and suicide risk assessment should be included in the curriculum.^15^

### Limitations

The opinions of the undergraduate students were not taken as a part of the needs analysis due to time constraints but the literature containing students’ perspectives was taken into consideration throughout the research.

## CONCLUSION

The current dental curriculum is not accommodating to the needs of the students in Peshawar. The current dental education environment lacks the infrastructure, logistics, and teacher training to sustain the standards set by PMDC.

### Authors’ Contribution:

**BM** conceived, designed, and did the analysis & writing of the manuscript.

**UM** helped in the conceptual design of the study, edited the manuscript, and gave final approval of the manuscript.

**BJ** did the review and final approval of the manuscript.

**NS** helped in the conceptual design of the project and the review of the manuscript.

All authors are responsible for the originality and integrity of research
